# Micro-costing and a cost-consequence analysis of the ‘Girls Active’ programme: A cluster randomised controlled trial

**DOI:** 10.1371/journal.pone.0221276

**Published:** 2019-08-16

**Authors:** Joanna M. Charles, Deirdre M. Harrington, Melanie J. Davies, Charlotte L. Edwardson, Trish Gorely, Danielle H. Bodicoat, Kamlesh Khunti, Lauren B. Sherar, Thomas Yates, Rhiannon Tudor Edwards

**Affiliations:** 1 Centre for Health Economics and Medicines Evaluation, Bangor University, Bangor, United Kingdom; 2 Diabetes Research Centre, University of Leicester, Leicester, United Kingdom; 3 Leicester Diabetes Centre, Leicester General Hospital, University Hospitals of Leicester, Leicester, United Kingdom; 4 Department of Nursing, School of Health, Social Care and Life Sciences, University of the Highlands and Islands, Inverness, United Kingdom; 5 School of Sport, Exercise and Health Sciences, Loughborough University, Loughborough, United Kingdom; The Open University, UNITED KINGDOM

## Abstract

Physical inactivity has been identified as a leading risk factor for premature mortality globally, and adolescents, in particular, have low physical activity levels. Schools have been identified as a setting to tackle physical inactivity. Economic evidence of school-based physical activity programmes is limited, and the costs of these programmes are not always collected in full. This paper describes a micro-costing and cost-consequence analysis of the ‘Girls Active’ secondary school-based programme as part of a cluster randomised controlled trial (RCT). Micro-costing and cost-consequence analyses were conducted using bespoke cost diaries and questionnaires to collect programme delivery information. Outcomes for the cost-consequence analysis included health-related quality of life measured by the Child Health Utility-9D (CHU-9D), primary care General Practitioner (GP) and school-based (school nurse and school counsellor) service use as part of a cluster RCT of the ‘Girls Active’ programme. Overall, 1,752 secondary pupils were recruited and a complete case sample of 997 participants (Intervention n = 570, Control n = 427) was used for the cost-consequence analysis. The micro-costing analysis demonstrated that, depending upon how the programme was delivered, ‘Girls Active’ costs ranged from £1,054 (£2 per pupil, per school year) to £3,489 (£7 per pupil, per school year). The least costly option was to absorb ‘Girls Active’ strictly within curriculum hours. The analysis demonstrated no effect for the programme for the three main outcomes of interest (health-related quality of life, physical activity and service use).Micro-costing analyses demonstrated the costs of delivering the ‘Girls Active’ programme, addressing a gap in the United Kingdom (UK) literature regarding economic evidence from school-based physical activity programmes. This paper provides recommendations for those gathering cost and service use data in school settings to supplement validated and objective measures, furthering economic research in this field.

**Trial registration:** -ISRCTN, ISRCTN10688342.

## Introduction

The World Health Organisation [1] identified a lack of physical activity as the fourth leading risk factor for global mortality, accounting for 6% of deaths globally. Physical activity levels in young people worldwide have been declining in recent years, with girls undertaking less physical activity than boys of the same age [2]. In the UK, recent data shows only 16% of 11–12 year old girls and 9% of 13–15 year old girls are sufficiently active [3]. Schools have been proposed as a setting to tackle inactivity, through the Physical Education (PE) curriculum, whole school campaigns, environment changes and the provision of after school clubs and activities [4–6]. There is a paucity of evidence for school-based physical activity interventions in the UK [7] and a lack of cost-effectiveness evidence globally surrounding physical activity interventions/programmes for children [8–9]. The lack of economic evidence surrounding this topic is problematic to agencies and local authorities in guiding decisions to deliver evidence-based interventions, which would also be considered a good use of resources. Local authorities and schools face difficult decisions about which services and resources to purchase against a backdrop of constrained and decreasing budgets. There is a need to implement effective programmes and, at the same time, for schools and local authorities to know and understand the budgetary implications of implementing such programmes.

Micro-costing involves collecting detailed information about the resources (human and financial) required to deliver the intervention/programme [10]. It is a bottom-up approach that assigns costs to each of the resource components [10]. In contrast, a top-down approach, also known as gross-costing, is calculated by dividing the total cost by the total number of units (i.e. total cost of the intervention/programme divided by the number of individuals receiving the intervention/programme) to obtain a mean cost of resource use [10]. Micro-costing is widely used in costing studies and is considered more accurate than gross-costing [11–13].

Cost-consequence analysis is a type of economic evaluation. Cost-consequence analysis presents, in a disaggregated form, an array of consequences/outcomes (e.g. health-related quality of life) and costs (e.g. health service use costs), in order for the decision maker to compare the two treatment arms of the trial (intervention group versus control group). The disaggregated format of the analysis does not combine costs and outcomes into a cost-effectiveness ratio or a cost-utility ratio. The verdict of whether or not the intervention would be considered a good use of resources is left to the decision maker [14–15]. Cost-consequence analysis is considered particularly relevant to economic evaluations undertaken alongside public health interventions [16]. Weatherly et al., [17] have argued for cost-consequence analyses to be conducted alongside cost-effectiveness or cost-utility analysis. This method allows researchers to assess multiple outcomes.

‘Girls Active’ was developed by the Youth Sport Trust (YST), the largest non-profit organisation focussing on youth sport and activity in the UK. ‘Girls Active’ is delivered in secondary schools and uses peer leadership and marketing to empower girls to take an active role in PE and sport provision within their school, and promote physical activity to peers. School leads are provided with resources and training to review their current PE and sport provision and ensure it is attractive to female pupils. The school leads then consult with female pupils to understand what changes could be undertaken to increase the relevance of physical activity provision to their peers. These changes are documented and revisited in an action plan and undertaken with the support of a hub school (a school with experience of targeting girls’ activity levels) if necessary.

The aim of this paper was to conduct a micro-costing and cost-consequence analysis of the ‘Girls Active’ programme, addressing the need for economic evidence of school-based physical activity interventions.

## Materials and methods

### Main cluster randomised controlled trial

A full description of the methods and results of the ‘Girls Active’ trial are available elsewhere [18–19]. Twenty secondary schools from the Midlands, UK were recruited to the two-arm cluster RCT. Ethical approval including the opt-out consent was obtained from the University of Leicester’s College of Medicine and Biological Sciences Research Ethics representative. A random sample of girls aged 11–14 years from each school were recruited. Following baseline measurements, schools were randomised by an independent statistician to one of the two groups, intervention or control (1:1) stratified by school size (pupil median: <850, ≥850) and percentage of black and minority ethnicity (BME) pupils (median: <20%, ≥20%). Ten schools received ‘Girls Active’ and 10 continued with usual practice. Measurements were conducted at baseline, 7 and 14 months. The primary outcome was objectively measured moderate- to vigorous-intensity physical activity (MVPA), measured by a 7-day wrist worn GENEActiv accelerometer at 14 months.

### The intervention–the ‘Girls Active’ programme

‘Girls Active’ provided a support framework for schools to review and change their physical activity, PE and sport provision, culture and practices. Training was provided prior to programme delivery, with an additional peer review day mid-way through the trial. Following training provided by YST, schools reviewed their current PE and sport provision. The student voice was encouraged and teachers actively sought student opinion on PE and sport within the school. A key element of the programme was for lead teachers in intervention schools to form a girls’ leadership and peer marketing group. The aim of this group was to empower girls to influence PE, sport and physical activity in their school, increase their own participation, develop as role models, and promote and market PE and sport to other girls. Lead teachers were offered in-person or telephone support through a hub school—and from the YST. They were also provided with two instalments of capacity funding (totalling £1,000) to assist with providing new activities requested by peer leaders and female pupils. The programme was delivered in the same manner as would have been done in the real-world setting.

#### Control condition

In comparison, control arm schools were not given any specific guidance or advice and were assumed to carry on with their usual practice of physical activity, PE and sport provision.

#### Micro-costing methods

Micro-costing methodology [10,12] was applied to calculate the costs of delivering the programme over a whole school year for the intervention schools taking part in the trial, to provide a mean cost per year, per school and per pupil. Per pupil costs were based on 467 pupils per school, in accordance with the average number of female pupils per school, taken from census data from all schools taking part in the trial (n = 20). In this micro-costing, we fully costed the delivery of the ‘Girls Active’ programme and its associated costs such as teacher time, travel and materials used. Costs were collected from a Local Education Authority perspective, accounting for on-costs (e.g. national insurance and pension costs), and using the cost year 2015–16.

A bespoke cost diary was designed and administered by one member of the research team to lead teachers responsible for delivering ‘Girls Active’. The diary asked lead teachers to complete a record of the additional time, or displaced time taken to offer the ‘Girls Active’ programme, and described activities undertaken and items purchased (e.g. sports equipment or clothing such as hoodies). The diary was developed with input from the wider ‘Girls Active’ research team and the YST who were responsible for training and supporting teachers during programme delivery.

Three versions of the diary were used throughout the course of the trial. The diary was originally sent to teachers via e-mail as a Microsoft Excel file, with sections for categories of programme activity (e.g. training, peer review day and programme delivery). Each section had a pre-written description of programme activity per row (e.g. time spent reviewing current PE and sport culture and practice (hours)), based on key programme components and delivery. The diary also had an ‘other’ section, which provided space for teachers to record programme activities and costs not covered by the sections/rows above. The diary was sent with written guidance for completion and presented to the lead teachers at the initial training event with a rationale and guidance for completion. An example of a completed diary sheet to provide the respondents with the information and level of detail required when completing the diary was also provided. We asked the teachers to complete the diary weekly. A member of the research team requested the Excel diary at two-month intervals whilst ‘Girls Active’ was being implemented (April 2015 until June 2016). Any queries regarding information provided were e-mailed to staff, and researcher contact details were provided in e-mails. After receiving the final Excel diary, any final queries were sent from the health economics researcher (JC) to the lead teacher who sent the diary via e-mail.

Over the first three months of the trial, the research team received feedback that around half of the teachers were not comfortable using the computer-based (Excel) format of recording costs. Teachers reported not being familiar or able to use Excel, and difficulties finding time to log into their e-mail or files to retrieve and add to the Excel. In response, a paper-based survey-style questionnaire was produced in Microsoft Word by two of the research team members (JC and DH). The survey used fields from the Excel diary, and the research team followed up surveys with a telephone call, from the researcher (JC) responsible for the micro-costing, to provide context to the responses or clarify. The survey was presented to the lead teachers who attended the peer leader event (January 2016). The event was organised by the hub school and the YST as an opportunity for the peer leaders to meet and share ideas. As well as commenting on the survey, the teachers stated that a simple logbook would be their preference as a simpler, easier-to-access format for recording time and costs. At the event, the teachers and the research team member (DH) in attendance co-produced the logbook with the lead teachers stating what headings they wanted in the logbook and how they would like it presented. The logbooks contained headings and row for activities and their associated time or cost, so teachers could record the information as it happened. To ensure we had cost information from as many schools as possible, the logbooks were printed and distributed to teachers by a member of the YST. It was requested that teachers keep these logbooks with them in their own diaries, and noted activity and costs related to ‘Girls Active’ as and when it happened. Each of the three methods requested demographic information about the teacher completing the diary including the school name, job title and salary band.

The salary band information was used to calculate teacher costs in the micro-costing, using national sources of salary costs. Teacher costs were sourced from the National Union of Teachers (NUT) pay structure for qualified classroom teachers in England and Wales (from 1 September 2015, for cost year 2015–2016) [20]. A school year consisting of 39 weeks was used to calculate the cost per hour for teacher costs, taking sickness, continuing professional development (CPD) and annual leave into account. Salary calculations were inclusive of employers’ on-costs. On-costs include National Insurance (NI) and Pension charges, as well as costs for annual increments and allowances. This mean hourly rate was applied to information supplied by teachers completing cost diaries. A school year of 39 weeks was chosen despite the variable time-periods of programme delivery in intervention schools in the trial to provide consistency in estimates of costs, and provide those interested in delivering the programme in the future a clear time horizon of costing. ‘Girls Active’ aimed to encourage a culture shift in schools, thus the principles of the programme would be expected to be undertaken annually and for school leads to be constantly ensuring their PE, physical activity and sport provision was appealing and of interest to pupils. Costs relating to research (e.g. time to complete diary, measures or undertake interviews) were not included in the final micro-costing calculations. This decision was made in order to provide local authorities with information pertinent for future rollout (training and delivery costs), rather than costs specific to conducting a research trial. Given the range of methods used to gather cost information, the following assumption was made during micro-costing in order to standardise the costing: Programme activities reported such as curriculum sessions, lunchtime sessions, and after school sessions were assumed to take place every week, throughout the academic year consisting of 39 weeks. These assumptions were made to provide consistency in estimates of costs and standardise a delivery approach in order to calculate costs. We note that intervention schools did not necessarily implement activities for this length of time.

Bottom-up micro-costing was chosen instead of top-down micro-costing, as the intervention was not prescriptive; it did not dictate the amount of time or money schools should spend to adapt their PE curriculum, it only stated core activities that should be conducted such as training, reviewing current activity and setting up a peer leader group. Given this intervention format, discrete information about resources and time taken to deliver ‘Girls Active’ was required from each of the intervention schools.

#### Cost-consequence methods

The cost-consequence analysis was conducted from a public sector, multi-agency perspective (community care, GP and local authority, school). Minutes of moderate-to-vigorous (MVPA) and health-related quality of life (CHU-9D) [21] were used as the measures of effect, and primary care (GP) and school-based services (school nurse and school counsellor) was the measure of costs. These health professionals were chosen in line with the perspective of the analysis and to reduce the burden on participants by enquiring about key health professionals pupils would be likely to come into contact within school and in the community.

Data was collected at baseline, approximately 7 months post-baseline and approximately 14 months post-baseline. The outcomes of interest for the cost-consequence analysis were mean minutes of MVPA per day, health-related quality of life measured by the CHU-9D [21] and use of primary care and school-based services. Mean minutes of MVPA per day were taken from data collected by the GENEActiv Original accelerometer (Activinsights Ltd, Kimbolton, UK) for worn 24 hours a day for 7 days by all pupils in the trial on their non-dominant wrist. In order to test uncertainty and as the follow-up period was more than 1 year, costs at 14-month post-baseline were discounted at the base rate of 3.5% [22] as part of sensitivity analyses. Outcomes were not discounted as part of the sensitivity analysis, given only an additional two months fell outside of the 1-year time horizon.

Pupils also completed two further questionnaires for the purposes of the economic evaluation. The CHU-9D [21] is a paediatric generic preference-based measure of health-related quality of life and consists of nine dimensions (worried, sad, pain, tired, annoyed, school work/homework, sleep, daily routine and ability to join in activities). The scores from each domain have a weighting applied and all domain weightings are summed together to produce a utility index. The CHU-9D has been validated with children aged 11 to 17 years as a self-report measure [21]. Participants were given specific guidance on how to complete this questionnaire. A list of common questions were collated and given to team members administering the questionnaires to participants to ensure a standardised response to any participant query. Documentation related to this study can be found at www.leicesterdiabetescentre.org.uk/girls-active-evaluation including a standardised operating procedure for administering the ‘Girls Active’ questionnaires.

The Client Service Receipt Inventory (CSRI) [23] is a questionnaire for collecting retrospective information about study participants’ use of health and social care services. The CSRI was administered at all time points, each time asking the participant to recall service use over the previous 7 months. This information was combined with national sources of reference unit costs [24] in order to calculate a mean cost of service costs per participant per arm for the cost consequences analysis. A supporting information file [S1 Table] shows a unit cost table outlining the published unit costs used in this cost-consequence analysis and their sources. The cost year of 2015–2016 was applied for all costs in UK Pounds Sterling.

#### Analysis

In order to analyse clustered data, we aligned the health economics analysis to the statistical analysis plan of the main trial, and used Generalised Estimating Equations (GEE), xtgee command in Stata. The GEE model was used to determine the difference between groups in change in mean MVPA minutes/day, CHU-9D [21] utility index scores and service use frequencies and costs between pupils from schools allocated to the programme and those allocated to control whilst taking account of clustering amongst pupils from the same school. The analysis included: two levels of clustering (pupil level and school level); a binary indicator for randomisation group as the explanatory variable; stratification categories based on school size of small vs. large (pupil median value: <850, ≥850), and percentage BME pupils low vs. high (pupil median percentage: non-white <20%, ≥20%) as potential confounders, adjustments for wear time (14-month measurement subtracted by the baseline) and final adjustments for the baseline measure of the outcome (MVPA).

The GEE model specification also included: an identity link function relating the mean response to the regression equation; Gaussian family distribution assumed for the response; an exchangeable correlation structure, which specifies the within-group correlation structure, and robust standard errors (SE) to provide consistent (i.e. asymptotically unbiased) parameter and SE estimates.

For service use frequency and cost models, GLM diagnostic tests were conducted as the models failed to converge using the above structure. From the results of the diagnostic tests, the family was amended from Gaussian to gamma and the link amended from identity to power -1. After changing these specific parameters, the models achieved convergence and these models were used for the variables of service use frequencies and costs in the xtgee models. For all three outcomes of interest the differences in marginal means the groups was calculated, and 95% Confidence Intervals (CIs) around these differences were produced with 1,000 bootstrapped replications.

## Results and discussion

### Summary of main findings from the ‘Girls Active’ cluster randomised trial

Harrington et al., [19] found no evidence of effect on the primary outcome of MVPA between intervention and control groups after 14 months; however, significant differences in MVPA were observed between groups at the shorter follow-up period of seven months. Subgroup analyses showed a significant effect for the programme in larger schools at 14 months. At seven months an effect for the programme was found for White Europeans and early maturers. Published methods to estimate biological maturity were used to calculate age at peak height velocity (APHV) [25] and maturation category [26]. Girls with an APHV < 1 standard deviation (SD) were classified as early maturers [26].

Sensitivity analyses showed similar results to the main analysis with no differences between groups at 14 months when the levels of accelerometer data were varied in the analyses. Differences in sub-groups may mean the programme could be targeted in future to certain types of schools or pupils. Given there was no effect found for the intervention based on the primary outcome (mean MVPA minutes/day), health-related quality of life and service use after 14 months, a cost-consequence analysis was conducted.

#### Micro-costing results

We received nine diaries/adequate information to generate costs for the schools in the intervention group. Out of the nine schools, four completed the Excel diary weekly. The remaining five schools provided their information through the survey. Three of the five teachers who completed a survey provided data in logbooks when returning the survey.

From the descriptions of the activity in the diaries and when contacting teachers, it was clear that the extent and methods of implementation varied across the nine schools as well as the length of time activities were delivered as part of the programme. Due to the nature of the programme, some of which was not prescriptive and gave schools the responsibility to implement the programme as appropriate within their own setting, ‘Girls Active’ was delivered in different ways, different extents and over different time-periods by the intervention schools taking part. In order to reflect the diversity of delivery, the results of the micro-costing are presented as three costings:

1. Within curriculum delivery–which entailed creating new development plans and delivering the programme within the curriculum after engaging with female pupils (base case).
2. Within curriculum and after-school delivery–which entailed creating new development plans and delivering the programme within the curriculum after engaging with female pupils, and included additional activity such as after-school clubs and taster sessions (approximately 3 hours per week).
3. Within curriculum, after-school delivery and day trips and events–which entailed creating new development plans and delivering the programme within the curriculum after engaging with female pupils, and included additional activity such as after-school clubs and day trips and events (approximately 4 hours per week and 4 trips per year, respectively).

The nine sets of diaries received were classified into the categories as thus: three schools were categorised as delivering within curriculum; four as delivering within curriculum and after-school, and finally two as delivering within curriculum, after-school and day trips and events. The mean time based on the schools in each category is presented in Table 1. A mean hourly rate of £26.00 (rounded to nearest pound) was applied in the micro-costing, which is based on the mean hourly rate of the salary information provided by the teachers completing the diaries.

**Table 1:** Costs of the programme based on the three delivery models reported by teachers in the trial.

	Within curriculum delivery (base case)	Within curriculum and after-school delivery	Within curriculum, after-school delivery and day trips and events
Type of cost	Units and costs^±^	Units and costs^±^	Units and costs^±^
All costs (non-recurrent and recurrent)			
Initial Training and initial resource costs			
Training course	One-off training course lasting 7 hours. £0.00 provided by YST as part of programme	One-off training course lasting 7 hours. £0.00 provided by YST as part of programme	One-off training course lasting 7 hours. £0.00 provided by YST as part of programme
Staff costs for a member of staff to attend training course	One-off training course lasting 7 hours for one teacher 7 hours x £26.00 = £182.00	One-off training course lasting 7 hours for one teacher 7 hours x £26.00 = £182.00	One-off training course lasting 7 hours for one teacher 7 hours x £26.00 = £182.00
Travel costs for a member of staff to attend training course	Mean distance travelled 22 miles 22 miles x 0.45 per mile = £10.00	Mean distance travelled 26.5 miles 26.5 miles x 0.45 per mile = £12.00	Mean distance travelled 44 miles 44 miles x 0.45 per mile = £20.00
Resources for schools including marketing plans, an action planning guide, branding toolkit	Resource pack describing ‘Girls Active’ £0.00 provided to schools by YST as part of programme	Resource pack describing ‘Girls Active’ £0.00 provided to schools by YST as part of programme	Resource pack describing ‘Girls Active’ £0.00 provided to schools by YST as part of programme
Peer Review Event			
Peer review event	One-off event lasting 7 hours £0.00 provided by Hub School and YST as part of programme	One-off event lasting 7 hours £0.00 provided by Hub school and YST as part of programme	One-off event lasting 7 hours £0.00 provided by Hub school and YST as part of programme
Teacher time spent at peer review event (hours)	4 hours x £26.00 = £104.00	4 hours x £26.00 = £104.00	4 hours x £26.00 = £104.00
Teacher travel distance to and from peer review event (miles)	Mean distance travelled 22 miles 22 miles x 0.45 per mile = £10.00	Mean distance travelled 26.5 miles 26.5 miles x 0.45 per mile = £12.00	Mean distance travelled 44 miles 44 miles x 0.45 per mile = £20.00
‘Girls Active’ school delivery costs^¥^			
Time spent reviewing current PE and sport culture and practice (hours)	2.5 hours x £26.00 = £65.00	8 hours x £26.00 = £208.00	5 hours x £26.00 = £130.00
Time spent recruiting ‘Girls Active’ leaders (hours)	2.25 hours x £26.00 = £59.00 spent recruiting a mean of 11 leaders	2.5 hours x £26.00 = £65.00 spent recruiting a mean of 11 leaders	3 hours x £26.00 = £78.00 spent recruiting a mean of 18 leaders
Time spent engaging with ‘Girls Active’ leaders to discuss current PE and school sport provision and what they would like to change (hours)	5 hours x £26.00 = £130.00 spent engaging with a mean of 11 leaders	10 hours x £26.00 = £260.00 spent engaging with a mean of 11 leaders	14 hours x £26.00 = £364.00 spent engaging with a mean of 18 leaders
Time spent engaging with pupils who are not ‘Girls Active’ leaders to discuss current PE and school sport provision and what they would like to change (hours)	5 hours x £26.00 = £130.00 spent engaging with a mean of 50 pupils	6 hours x £26.00 = £156.00 spent engaging with a mean of 130 pupils	4 hours x £26.00 = £104.00 spent engaging with a mean of 70 pupils
Time spent developing action plans (hours)	8 hours x £26.00 = £208.00	10 hours x £26.00 = £260.00	10 hours x £26.00 = £260.00
Time spent planning new activities in order to implement the action plans (hours)	4 hours x £26.00 = £104.00	8 hours x £26.00 = £208.00	9 hours x £26.00 = £234.00
Total time spent implementing and delivering ‘Girls Active’ per week from logbooks, surveys and diaries–including undertaking PE lessons, meeting with staff and ordering new equipment (hours)	7 hours* x 39 weeks = 273 hours*	7 hours* x 39 weeks = 273 hours*	7 hours* x 39 weeks = 273 hours*
After school sessions and delivered as part of ‘Girls Active’ per week	0 hours = £0.00	3 hours* x 39 weeks = 117 hours*	4 hours* x 39 weeks = 156 hours*
Day trips as part of delivering ‘Girls Active’ over the year	0 hours = £0.00	0 hours = £0.00	6 hours x mean of 4 trips over the year = 24 hours 24 hours x £26.00 = £624.00
Total amount of capacity building funding received to implement ‘Girls Active’ (£)	2 x £500 Capacity funding provided by YST for equipment £0.00 provided by YST	2 x £500 Capacity funding provided by YST for equipment £0.00 provided by YST	2 x £500 Capacity funding provided by YST for equipment £0.00 provided by YST
Additional costs for PE and school sport equipment in order to deliver the ‘Girls Active’ action plan–not covered by capacity building funding, instead supplied by school’s own funding (£)	£0.00	£900.00 for sporting equipment and awards	£1,300 for coach hire for day trips, additional coaching (e.g. gymnastics)
Time spent utilising support from Hub School (Crown Hills) and YST (including e-mail correspondence, telephone calls and face to face contact) (hours)	2 hours x £26.00 = £52.00	2 hours x £26.00 = £52.00	3 hours x £26.00 = £78.00
Total costs per school, per year^±^	£1,054.00	£2,419 .00	£3,498.00
Total costs per pupil, per school, per year (based on average of 467 female pupils per school, from census data from all 20 schools taking part in the trial)^±^	£1054/467 = £2.00	£2419/467 = £5.00	£3498/467 = £7.00

* undertaken as part of contractual “usual” hours.

± costs rounded to nearest pound.

^¥^ costs standardised using the assumption activities took place each week for a school year of 39 weeks.

Table 1 shows a range of costing models that could be employed to deliver the programme. Costs ranged from £1,054 (£2 per pupil, per school year) to £3,498 (£7 per pupil, per school year), with the least costly option being to absorb ‘Girls Active’ strictly in curriculum hours. Staff time for intervention delivery accounted for the largest costs in the micro-costing due to the nature of the intervention, which was not prescriptive. Teachers were not told the amount of time or money schools should spend to adapt their PE curriculum, which led to teachers deciding what resources and effort they should give to the intervention. Schools also received £1,000 of funding from the YST, though the majority of schools did not spend any of these funds during the trial. Those who spent some of the funding generally used the funding to hire external coaches or purchase new equipment in order to deliver activities requested by the pupils, for example, dodgeballs and Zumba kits. Some schools received further funding from their school’s own budget and public sector funding to further support the purchase of equipment and provision of new activities.

Opportunity costs were considered in the micro-costing. Opportunity cost is defined as the value of benefits foregone by not using resources in their next best alternative use. As ‘Girls Active’ was delivered in usual school hours replacing previous PE activities with those specified by the pupils, this resulted in minimal opportunity costs for the schools taking part in the trial. However, it is worth stating that in order to deliver ‘Girls Active’ teachers’ usual activities were sometimes displaced, this included Planning, Preparation and Assessment (PPA) time, overseeing non-‘Girls Active’ clubs and free periods as well as personal time outside of working hours. On average across the nine intervention schools included in the micro-costing analysis, teachers experienced 6 hours of displaced activity over the course of the year consisting of 39 weeks.

The three costings provide local authorities and those interested in funding the delivery of ‘Girls Active’ in the future an estimate of the time and resources used when the delivery models witnessed in this trial are extrapolated out to a full academic year (39 weeks). The costings do not make any claims on the quality of the delivery; a process evaluation of the programme is presented in a separate paper [27].

#### Health economics sample

In the trial, 1,752 participants were recruited. For the economics analysis, we excluded participants who had missing data for both costs and outcomes, as economic evaluations required complete cases from both costs and outcomes [14]. Of these 1,752 participants, 1,211 had primary outcome (mean MVPA minutes/day) data at both baseline and 14 months post-baseline. 1,163 had CHU-9D [21] data at both baseline and 14 months post-baseline, and 1,157 participants had service use data at both baseline and 14 months post-baseline. After missing data was removed, and participant IDs were matched across time-points, the final sample for the economic complete case analysis was N = 997 (Intervention n = 570, Control n = 427).

Table 2 summarises the characteristics of the complete case economic sample at baseline (N = 997), demonstrating the two groups were similar in terms of ethnicity, age in years, indices of multiple deprivation and proportion in each year group. Of the total sample, 21.5% were of non-white European background, the mean age was 13 years, with a low proportion of free school meal eligibility and classed as having mid-levels of deprivation as measured by the Department for Communities and Local Government English indices of deprivation deciles [28].

**Table 2:** Baseline characteristics of the economic sample (N = 997) and whole sample (N = 1,752), separated by randomisation arm^†^.

Characteristic	Economic Sample (N = 997)		Whole Sample (N = 1,752)	
	Intervention arm	Control arm	Intervention arm	Control arm
Participants (n)	570	427	867	885
Ethnic Group	n (%)	n (%)	n (%)	n (%)
White	467 (82)	319 (75)	673 (77)	669 (76)
Non-white	103 (18)	108 (25)	194 (23)	216 (24)
Year Group				
Year 7	206 (36)	194 (46)	318 (37)	356 (40)
Year 8	232 (41)	176 (41)	365 (42)	355 (40)
Year 9	132 (23)	57 (13)	184 (21)	174 (20)
	Mean (SD), range	Mean (SD), range	Mean (SD), range	Mean (SD), range
Age at baseline (years)	12.9 (0.8), 12–14	12.7 (0.8), 11–14	12.8 (0.8), 11–14	12.8 (0.8), 11–14
Pupils who classified their ethnicity as non-white per school (%)	22.5 (27.5), 1–88	29.6 (35.7), 5–96	21.6 (26.7), 1–88	24.9 (32.1), 5–96
Pupils eligible for free school meals per school (%)	12.7 (5.2), 7–27	10.2 (6.4), 4–21	13.3 (5.3), 7–27	9.6 (5.9), 4–21
School Index of Multiple Deprivation (IMD) (decile score)^±^	6.2 (2.4), 1–9	7.2 (2.4), 4–10	6.1 (2.4), 1–9	7.2 (2.2), 4–10
Pupil IMD (decile score)^±^	5.2 (2.7), 1–10	6.3 (3.0), 1–10	5.1 (2.7), 1–10	6.5 (2.8), 1–10

^†^ number of observations reduces to intervention (n = 532) and control (n = 407) for the economic analyses.

^±^ higher the number the least deprived.

Table 2 also shows the baseline characteristics of the whole sample (N = 1,752). It shows the complete case economic sample (N = 997) and the whole sample (N = 1,752) were similar in terms of ethnicity, age in years, indices of multiple deprivation and proportion in each year group.

Marginal means are presented throughout, as these report the mean following the xtgee model and take account of clustering. Table 3 show the results from the xtgee models, demonstrating the programme did not have an effect on the main outcomes of MVPA/day, CHU-9D [21] utility index score or total service use for the economic sample. However, factors such as baseline value, school size and percentage of ethnic minority pupils did have an effect on the results, adding weight to the trial design and therefore their inclusion in the xtgee model. Table 4 shows no significant differences between the means of the intervention and control group for the three outcome measures and costs as indicated by the bootstrapped CIs (bootstrapping was used to create 1,000 valid bootstrap replications). A supporting information file [S2 Table] shows the results of the xtgee model and marginal means of service use when broken down into individual services of GPs, school nurses and school counsellors at each time-point. The analysis found no statistically significant effect for the programme when service use was divided into individual services.

**Table 3:** Results from the xtgee models for the three economic outcomes*^±^.

Factors	MVPA/day 14 months post-baseline β coefficient, SE (95% CI)	CHU-9D utility index score at 14 months post-baseline β coefficient, SE (95% CI)	Total frequencies of service use at 14 months post-baseline β coefficient, SE (95% CI)	Total costs of service use at 14 months post-baseline β coefficient, SE (95% CI)
Randomisation	1.37, 1.54 (-1.64–4.38)	-0.01, 0.01 (-0.24–0.01)	0.04, 0.02 (-0.00–0.07)	-0.00, 0.00 (-0.00–0.00)
Minutes of MVPA/day at baseline	0.64, 0.38 (0.57–0.72)*	———-	———-	———-
CHU-9D utility index score at baseline	———-	0.58, 0.04 (0.52–0.65)*	———-	———-
Total frequencies of service use at baseline	———-	———-	-0.02, 0.00 (-0.03 –-0.02)*	———-
Total costs of service use at baseline	———-	———-	———-	-0.00, 9.97 (-0.00 –-0.00)*
School size (<850, ≥850 pupils)	-1.21, 1.99 (-5.11–2.70)	-0.00, 0.01 (-0.02–0.01)	0.16, 0.04 (0.09–0.23)*	0.00, 0.00 (-0.00–0.00)
Percentage of BME pupils (<20%, ≥20%)	-3.93, 1.19 (-6.27 –-1.59)*	0.03, 0.01 (0.01–0.04)*	-0.14, 0.02 (-0.18 –-0.10)*	0.00, 0.00 (-0.00–0.00)
Constant	14.49, 2.86 (8.89–20.08)*	0.32, 0.04 (0.25–0.39)*	0.41, 0.02 (0.36–0.45)*	0.01, 0.00 (0.01–0.01)*

* Significant at .05 significance level.

^±^ β coefficients, SEs and 95% CIs all rounded to 2 decimal places.

**Table 4:** Marginal mean minutes/day of MVPA, CHU-9D [21] utility index scores and total service use following 14-month post baseline xtgee models, and mean differences between groups (1,000 bootstrapped 95% CI)^±^.

Outcome	Marginal mean, SE (95% CI)		Difference between groups (1,000 bootstrapped 95% CI)
	Intervention (n = 570)	Control (n = 427)	
Minutes of MVPA/day	42.22, 0.83 (40.60–43.84)	41.69, 1.14 (39.46–43.93)	0.53 (0.00–0.00)
CHU-9D utility index score	0.82, 0.00 (0.81–0.83)	0.84, 0.01 (0.82–0.85)	0.01 (-0.02–0.00)
Total Service use frequency	2.43, 0.12 (2.20–2.66)	3.25, 0.37 (2.52–3.98)	——————-
Total Service use cost	94.32, 7.23 (80.14–108.49)	115.69, 22.85 (70.90–160.48)	21.38 (-151.92–123.41)

^±^ Marginal means, SEs and 95% CIs all rounded to 2 decimal places.

After conducting the xtgee models, inter cluster correlations (ICCs) were calculated for the three outcomes. An ICC of 0.198 was found for MVPA/day; -0.000 for CHU-9D [21] utility index score; -0.008 for frequencies of total service use; and -0.005 for costs of total service use, respectively.

#### Sensitivity analysis

As the follow-up period was more than one year, as part of sensitivity analysis, service use costs at 14-month post-baseline were discounted at 3.5%, the base rate recommended by NICE [16], and the model was re-run with these discounted costs. Table 5 demonstrates the sensitivity analyses replicated the main analysis results, showing no effect for the programme, but an effect of baseline costs and constant. Discounted marginal means reported in Table 5 are similar to the undiscounted marginal means reported in Table 4.

**Table 5:** Results from the xtgee models using discounted costs as part of sensitivity analyses*^±^.

Factors	Discounted total costs of service use at 14 months post-baseline β coefficient, SE (95% CI)		Discounted costs of GP service use at 14 months post-baseline β coefficient, SE (95% CI)		Discounted costs of school nurse service use at 14 months post-baseline β coefficient, SE (95% CI)		Discounted costs of school counsellor service use at 14 months post-baseline β coefficient, SE (95% CI)	
Randomisation	-0.00, 0.00 (-0.00–0.00)		-1.85, 4.83 (-11.31–7.61)		1.29, 5.25 (-9.01–11.59)		-4.67, 7.44 (-19.26–9.92)	
Total costs of service use at baseline	-0.00, 1.03 (-0.00 –-0.00)*		———-		———-		———-	
Costs of GP service use at baseline	———-		13.56, 2.73 (8.22–18.91)*		———-		———-	
Costs of school nurse service use at baseline	———-		———-		0.36, 0.08 (0.21–0.52)*		———-	
Costs of school counsellor service use at baseline	———-		———-		———-		0.47, 0.05 (0.38–0.57)*	
School size (<850, ≥850 pupils)	0.00, 0.00 (-0.00–0.00)		-9.82, 6.87 (-23.28–3.64)		-5.21, 6.73 (-18.41–7.99)		-5.40, 8.43 (-21.91–11.12)	
Percentage of BME pupils (<20%, ≥20%)	0.00, 0.00 (-0.00–0.00)		-3.82, 5.23 (-14.07–6.43)		10.61, 5.63 (-0.42–21.64)		5.96, 9.16 (-11.99–23.91)	
Constant	0.01, 0.00 (0.01–0.01)*		38.32, 4.95 (28.63–48.02)*		17.99, 7.75 (2.79–33.19)*		22.98, 9.24 (4.87–41.09)*	
				
	Intervention (n = 570)	Control (n = 427)	Intervention (n = 570)	Control (n = 427)	Intervention (n = 570)	Control (n = 427)	Intervention (n = 570)	Control (n = 427)
Marginal mean, SE (95% CI)	91.13, 6.99 (77.43–104.83)	111.78, 22.08 (68.50–155.06)	45.80, 3.53 (38.89–52.73)	49.12, 4.10 (41.10–57.15)	27.44, 2.78 (21.98–32.89)	26.93, 4.52 (18.08–35.79)	20.43, 4.95 (10.74–30.13)	30.57, 541 (19.96–41.17)

* Significant at .05 significance level.

^±^ Marginal mean, β coefficients, SEs and 95% CIs all rounded to 2 decimal places.

A supporting information file [S3 Table] shows the results of the exploratory sub-group analyses, testing the effects of year group and programme delivery model (based upon the levels described in the micro-costing). In this analysis, year group was found to have a statistically significant effect on minutes of MVPA per day, but did not affect any other outcomes. Programme delivery model did not affect the main outcomes of MVPA/per day, CHU9D [21] utility index scores and frequencies and costs of service use. No other statistically significant differences were found.

## Discussion

### Summary of findings

The cost-consequence analysis demonstrated, depending upon how ‘Girls Active’ was implemented, costs ranged from £1,054 (£2 per pupil, per school year) to £3,498 (£7 per pupil, per school year). There were no statistically significant differences found between the groups for the three outcomes of interest (MVPA/per day, CHU9D [21] utility index scores, and frequencies and costs of service use). However, factors such as scores on outcome measures at baseline, school size and percentage of ethnic minority pupils were shown to have an effect. Sensitivity analysis applying a 3.5% discount rate to costs at 14 months post-baseline showed no effect for the programme, replicating the main analysis results.

The ‘Girls Active’ trial with an embedded economic analysis showed both the intervention and control groups reported less than the recommended 60 minutes or more of MVPA per day for girls aged 5 to 17 years [1]. The mean CHU-9D [21] utility index scores of this sample were slightly lower than the mean score reported by Radcliffe et al., [29] in a UK community sample of 11 to 17 year olds. The intervention group reported lower mean frequencies and costs of service use than the control group. However, this difference was not statistically significant.

#### Strengths and limitations

Schools are viewed as an important setting for public health programmes [30]. This micro-costing and cost-consequence analysis adds to the limited literature and provides results from a UK context. By gathering individual information on programme delivery, we demonstrated the programme could be delivered using a range of delivery methods with a range of associated costs, and we also increased the likelihood of receiving data from each intervention school.

The study was limited by the amount of missing data, which reduced the sample for the economic evaluation, due to the need for complete cases. The main area of missing data came from service use. We endeavoured to focus on key professionals, to increase relevance and reduce burden. Future trials of school-based interventions including a health economics component could shorten the recall period, ask parents to complete the measure or explore the use of electronic records.

We acknowledge the limitations in collecting costs using three different methods, and note that by assuming a school year of 39 weeks and consistent delivery of activities related to the ‘Girls Active’ programme over this year, our costings might have overestimated the costs of the programme [27]. We tried to reduce over-estimation as much as possible by not costing activities that would have taken place in contractual hours and using the information provided by intervention schools to give a reflection of the length of time teachers spent on activities such as specific lessons, after school clubs and day trips. These assumptions were made to standardise costing across schools and provide clear costing for those interested in delivering the programme in future.

#### Novel aspects of applying health economics to a school-based physical activity trial

Conducting economic evaluations alongside public health interventions has been described as challenging, often requiring a pragmatic approach [31, 17]. The difficulties faced by teachers in completing the Excel version of the cost diary, which was essential for the economics analysis to quantify the resources required to the deliver the ‘Girls Active’ programme, raised a particular challenge in this trial early on. A pragmatic and iterative approach to co-designing measures to collect this information was taken, adapting the Excel cost diary to a survey and listening to feedback by creating a logbook to increase response rates. Teachers were followed up with telephone calls to gain all information required, and used assumptions to provide consistency across the three different methods employed. This adaptive approach to the economic data collection yielded further responses from the teachers, and resulted in 9 out of 10 intervention schools providing data for the micro-costing analysis.

The ‘Girls Active’ programme is not prescriptive in nature. The programme consisted of a number of core steps that all lead teachers were asked to attend or do. This included attending training and peer review events, completing a school self-review and submitting two action plans, and setting up a peer leader group. However, it did not dictate how schools should implement activities within their own school, how the student voices should be used or what the capacity building funding should be spent on; although, examples and suggestions were given by the YST at each of the training events and through the development coach. The programme did not state the number of hours teachers should spend recruiting peer leaders, reviewing current activity and developing new lesson plans. This presented the challenge of displaying the costs of the programme in a meaningful way that reflected what happened for the intervention schools taking part in the trial, but could also be used by future local authorities to indicate the cost of ‘Girls Active’ if it is to be rolled out in their locality. Due to the adaptive approach taken to collecting cost information, we increased the amount of data available to draw from. Furthermore, by undertaking follow-up telephone calls with the teachers who completed the cost diaries, we had first-hand accounts of their experiences, which provided context to the information received. This context was instrumental in helping the researchers to make the decision to present the data as three costings, using the different delivery methods, which we believe provides useful information for those interested in delivering ‘Girls Active’ in a real-world setting. However, assumptions had to be made in the micro-costing to standardise delivery across the intervention schools.

#### Lessons learned—Implications for practice and/or future research

The cost-consequence analysis quantified the resources used in the trial to undertake differing levels of implementation to deliver the programme. However, in trials conducted in the community there is a balance to be achieved between quantity and quality of data. Given our previous experiences [32–33], weekly Excel diaries were considered the preferred method to capture programme delivery information as they use a shorter recall period and could be used to track activity as and when it happened. However, the trial demonstrated this approach does not seem to fit with the school context or how teachers operate during a school day; teachers in the trial most preferred the surveys. In future trials, it would be beneficial to undertake consultations with the teachers prior to the start of a trial to find a method to capture costs that works for both the teachers and researchers. It would also negate the need to adapt and develop methods through the course of the trial, which could be burdensome and confusing for those completing them. However, given the experiences of the researchers in the trial we would advise others undertaking research in school settings to consider alternatives if prechosen methods prove unsuccessful. There is also a need to align questioning with other components of the research, for example, qualitative research components. The micro-costing and process evaluation of the trial found disparity in information provided by teachers [27]. This could be attributed to the line of questioning used by each component and the timing of the interviews compared to the follow-up telephone calls.

Service use information was difficult to obtain in the trial from participants. Though the measure was kept brief, the seven-month period of recall seemed to be the main source of difficulty. The difficulty in remembering contacts could also be attributed to the relative levels of independence and autonomy in the sample, whose ages ranged from 11 to 14 years old. Though adolescence is considered a time when individuals are experiencing greater independence and autonomy, it is likely that for many of the sample appointments such as those to see a GP would be arranged by a parent or guardian. It is also likely that the parent/guardian would remind their child of the appointment and take them to the surgery, thus perhaps making it harder for participants to recall contacts with the healthcare professionals when asked. Future research could use parental recall rather than pupil recall or explore the use of electronic records.

The trial used an objective measure of physical activity. Though this is a strength of the trial as previous research has criticised the reliance of self-report measures in physical activity research [34–36], the trial could have been further strengthened by quantifying attendance rates, numbers of sick notes and participation of pupils who usually have no interest or enjoyment in PE, sport and physical activity as part of the main outcomes. This additional information could have been used to assess the culture shift in schools, which is a key driver of the ‘Girls Active’ programme. Given the approach of the ‘Girls Active’ programme, which focuses upon empowerment and encouragment in physical activity, sport and PE, inclusion of this information could help ascertain if pupils who would have previously avoided PE and sport using absence, sick notes and non-participation begin to attend lessons, join in and take more of an active role in games and sports. These outcomes would align more with the ethos of the programme, and would provide useful additional data alongside validated objective measures such as accelorometers.

## Conclusions

This paper reports a micro-costing and cost-consequence analysis of a school-based physical activity programme for girls, answering a need for further economic evidence in this field, particularly in a UK context. The trial provides useful costing information for local authorities interested in the programmes, which addresses a gap in UK knowledge. The paper also provides lessons for those conducting research in school settings concerning gathering detailed information on programme delivery, aligning different research components, collecting service use information and use of data describing participation in physical activity, sport and PE to supplement validated and objective measures, furthering economic evaluations in this field, which is particularly important given the current limited evidence base.

## Supporting information

S1 Table Unit cost table outlining the published unit costs used in this cost-consequence analysis and their sources. (DOCX)

S2 Table Results of the xtgee model and marginal means of service use when broken down into individual services of GPs, school nurses and school counsellors at each time-point. (DOCX)

S3 Table Results of the exploratory sub-group analyses, testing the effects of year group and programme delivery model (based upon the levels described in the micro-costing). (DOCX)

## Abbreviations

BME: Black and minority ethnicity
CI: Confidence interval
GP: General Practitioner
ICC: Intra-class correlation
IMD: Index of multiple deprivation
MVPA: Moderate- to vigorous-intensity physical activity
NHS: National Health Service
PE: Physical education
RCT: Randomised controlled trial
SE: Standard error
SD: Standard deviation
UK: United Kingdom
YST: Youth Sport Trust
