# Supplementary material for: Micro-costing and a cost-consequence analysis of the ‘Girls Active’ programme: A cluster randomised controlled trial
Source: PLoS One. 2019 Aug 16;14(8):e0221276. doi: 10.1371/journal.pone.0221276 (PMC6697369; doi:10.1371/journal.pone.0221276)
Supplement: S1 Table — (DOCX) [file pone.0221276.s001.docx]

Additional File 1. Table 1. Unit costs of service use in UK pounds for cost year 2015-2016 with sources^a^

| Health-care resource | Unit | Unit cost (£) | Details and Source |
| --- | --- | --- | --- |
| Primary Health Care and Community Services | | | |
| GP (Clinic) | Visit | 36 | Per surgery consultation lasting 9.22 minutes, including direct care staff costs and with qualifications^b^ |
| School Nurse or specialist nurse | Visit | 44 | Per consultation lasting 30minutes (Curtis 2007)^c^ |
| District Nurse | Visit | 42 | Per working hour with qualifications (Band 6 Counsellor)^b^ |

^a^ NHS costs to nearest pound, including salary, employers’ costs, overheads and capital costs.

^b^ From Curtis & Burns (2016)

^c^ From Bywater et al (2009) and inflated to cost year 2015/16 using Curtis and Burns (2016) Hospital Pay and Prices Inflation Indices.
