# Supplementary material for: Micro-costing and a cost-consequence analysis of the ‘Girls Active’ programme: A cluster randomised controlled trial
Source: PLoS One. 2019 Aug 16;14(8):e0221276. doi: 10.1371/journal.pone.0221276 (PMC6697369; doi:10.1371/journal.pone.0221276)
Supplement: S2 Table — (DOCX) [file pone.0221276.s002.docx]

Additional File 2. Table 1 Results from the xtgee models of individual services^*±^

| Factors | Frequencies of GP service use at 14-months post-baseline^±^ | Costs of GP service use at 14-months post-baseline^±^ | Frequencies of school nurse service use at 14-months post-baseline^±^ | Costs of school nurse service use at 14-months post-baseline^±^ | Frequencies of school counsellor service use at 14-months post-baseline^±^ | Costs of school counsellor service use at 14-months post-baseline^±^ |
| --- | --- | --- | --- | --- | --- | --- |
| Randomisation | -0.06, 0.14  (-0.33 – 0.22) | -2.02, 4.97  (-11.76 – 7.73) | 0.03, 0.12  (-0.21 – 0.27) | 1.34, 5.44  (-9.32 – 12.00) | -0.12, 0.18  (-0.48 – 0.24) | -4.83, 7.70  (-19.93 – 10.27) |
| Frequencies of GP service use at baseline | 0.39, 0.08  (0.24 – 0.54)* | ---------- | ---------- | ---------- | ---------- | ---------- |
| Costs of GP service use at baseline | ---------- | 0.39, 0.08  (0.24 – 0.54)* | ---------- | ---------- | ---------- | ---------- |
| Frequencies of school nurse service use at baseline | ---------- | ---------- | 0.38, 0.08  (0.21 – 0.54)* | ---------- | ---------- | ---------- |
| Costs of school nurse service use at baseline | ---------- | ---------- | ---------- | 0.38, 0.08  (0.21 – 0.54)* | ---------- | ---------- |
| Frequencies of school counsellor service use at baseline | ---------- | ---------- | ---------- |  | 0.49, 0.05  (0.39 – 0.58)* | ---------- |
| Costs of school counsellor service use at baseline | ---------- | ---------- | ---------- | ---------- | ---------- | 0.49, 0.05  (0.39 – 0.58)* |
| School size (<850, ≥850 pupils) | -0.28, 0.20  (-0.66 – 0.11) | -10.26, 7.08  (-24.13 – 3.61) | -0.12, 0.16  (-043 – 0.19) | -5.39, 6.97  (-19.05 – 8.26) | -0.13, 0.21  (-0.54 – 0.27) | -5.59, 8.72  (-22.68 – 11.51) |
| Percentage of BME pupils (<20%, ≥20%) | -0.12, 0.15  (-0.41 – 0.18) | -3.84, 5.39  (-14.39 – 6.72) | 0.25, 0.13  (-0.01 – 0.51) | 10.98, 5.82  (-0.44 – 22.40) | 0.15, 0.23  (-0.29 – 0.60) | 6.17, 9.48  (-12.41 – 24.74) |
| Constant | 1.10, 0.14  (0.82 – 1.38)* | 39.73, 5.12  (29.70 – 49.76)* | 0.42, 0.18  (0.07 – 0.78)* | 18.62, 8.03  (2.89 – 34.35)* | 0.57, 0.23  (0.12 – 1.01) | 23.78, 9.56  (5.04 – 42.53)* |

* Significant at .05 significance level

^±^ All values reported are β coefficient, SE (95% confidence interval). All values all rounded to 2 decimal places.

Table 2. Marginal means of individual services following 14-month post baseline xtgee models^±^.

| Outcome | Marginal mean, SE (95% Confidence Interval) | |
| --- | --- | --- |
|  | Intervention  (n = 570) | Control  (n = 427) |
| Frequencies of GP service use | 1.32, 0.10 (1.12 – 1.52) | 1.42, 0.12 (1.19 – 1.65) |
| Costs of GP service use | 47.32, 3.64 (40.19 – 54.45) | 50.81, 4.22 (42.53 – 59.09) |
| Frequencies of school nurse service use | 0.65, 0.07 (0.52 – 0.77) | 0.64, 0.17 (0.43 – 0.85) |
| Costs of school nurse service use | 28.40, 2.89 (22.75 – 34.04) | 27.88, 4.68 (18.71 – 37.04) |
| Frequencies of school counsellor service use | 0.50, 0.12 (0.27 – 0.74) | 0.75, 0.13 (0.49 – 1.02) |
| Costs of school counsellor service use | 21.15, 5.12 (11.11 – 31.18) | 31.64, 5.60 (20.66 – 42.61) |

^±^ Marginal means, SEs and 95% Confidence Intervals all rounded to 2 decimal places.
