# Supplementary material for: Micro-costing and a cost-consequence analysis of the ‘Girls Active’ programme: A cluster randomised controlled trial
Source: PLoS One. 2019 Aug 16;14(8):e0221276. doi: 10.1371/journal.pone.0221276 (PMC6697369; doi:10.1371/journal.pone.0221276)
Supplement: S3 Table — (DOCX) [file pone.0221276.s003.docx]

Additional File 3. Table 1 Results from the xtgee models including the variables of year group and programme delivery model as part of exploratory sub-group analyses*±

| Factors | MVPA/day 14-months post-baseline  β coefficient, SE (95% confidence interval) | CHU-9D utility score at 14-months post-baseline  β coefficient, SE (95% confidence interval) | Total frequencies of service use at 14-months post-baseline  β coefficient, SE (95% confidence interval) | Total costs of service use at 14-months post-baseline  β coefficient, SE (95% confidence interval) |
| --- | --- | --- | --- | --- |
| Randomisation | 4.76, 2.72 (-0.56 – 10.08) | -0.01, 0.13 (-0.03 – 0.02) | -0.04, 0.06 (-0.07 – 0.15) | -0.00, 0.00 (-0.00 – 0.00) |
| Minutes of MVPA/day at baseline | 0.63, 0.37 (0.55 – 0.70)* | ---------- | ---------- | ---------- |
| CHU-9D utility index score at baseline | ---------- | 0.59, 0.03 (0.52 – 0.65)* | ---------- | ---------- |
| Total frequencies of service use at baseline | ---------- | ---------- | -0.02, 0.00 (-0.03 – -0.02)* | ---------- |
| Total costs of service use at baseline | ---------- | ---------- | ---------- | -0.00, 1.49 (-0.00 – -0.00)* |
| School size (<850, ≥850 pupils) | -2.54, 2.48 (-7.40 – 2.31) | -0.00, 0.01 (-0.02 – 0.02) | 0.02, 0.03 (-0.08 – 0.05)* | -0.00, 0.00 (-0.00 – 0.00) |
| Percentage of BME pupils (<20%, ≥20%) | -4.03, 1.10 (-6.19 – -1.88)* | 0.03, 0.01 (0.01 – 0.04)* | -0.06, 0.02 (-0.11 – -0.01)* | -0.00, 0.00 (-0.00 – -0.00)* |
| Year group | -1.92, 0.61 (-3.11 - -0.72)* | 0.00, 0.01 (-0.01 – 0.01) | 0.02, 0.02 (-0.03 – 0.07) | 0.00, 0.00 (-0.00 – 0.00) |
| Programme delivery model (based on micro-costing categories of; Within curriculum delivery (base case); Within curriculum and after-school delivery; Within curriculum, after-school, day trips and events delivery. | -1.79, 1.34 (-4.42 – 0.85) | 0.00, 0.01 (-0.01 – 0.01) | -0.04, 0.03 (-0.10 – 0.02) | -0.00, 0.00 (-0.00 – 0.00) |
| Constant | 17.65, 3.05 (11.67 – 23.63)* | 0.31, 0.03 (0.25 – 0.38)* | 0.51, 0.05 (0.42 – 0.61)* | 0.01, 0.00 (0.01 – 0.02)* |

* Significant at .05 significance level

± Marginal mean, β coefficients, SEs and 95% Confidence Intervals all rounded to 2 decimal places.

Table 2 marginal means of MVPA/day, CHU-9D (Stevens, 2011) utility index score and frequencies and costs of service use split by year group and programme delivery model following xtgee models±.

|  | MVPA/day 14-months post-baseline  Marginal mean, SE (95% confidence interval) | | CHU-9D utility score at 14-months post-baseline β coefficient, SE (95% confidence interval) | | Total frequencies of service use at 14-months post-baseline  β coefficient, SE (95% confidence interval) | | Total costs of service use at 14-months post-baseline  β coefficient, SE (95% confidence interval) | |
| --- | --- | --- | --- | --- | --- | --- | --- | --- |
| Year 7 | Intervention  (n = 206) | Control  (n = 194) | Intervention  (n = 206) | Control  (n = 194) | Intervention  (n = 206) | Control  (n = 194) | Intervention  (n = 206) | Control  (n = 194) |
|  | 47.19, 1.44  (44.36 – 50.02) | 46.07, 1.22 (43.67 – 48.46) | 0.81, 0.01  (0.80 – 0.83) | 0.85, 0.01  (0.83 – 0.86) | 2.62, 0.33  (1.98 – 3.26) | 2.54, 0.19  (2.17 – 2.91) | 104.41, 13.44 (78.07 – 130.76) | 100.78, 7.84  (85.41 – 116.15) |
| Year 8 | Intervention  (n = 232) | Control  (n = 176) | Intervention  (n = 232) | Control  (n = 176) | Intervention  (n = 232) | Control  (n = 176) | Intervention  (n = 232) | Control  (n = 176) |
|  | 41.31, 1.25  (38.86 – 43.77) | 39.75, 1.70 (36.41 – 43.09) | 0.83, 0.01  (0.82 – 0.84) | 0.83, 0.01  (0.81 – 0.86) | 2.16, 0.33  (1.51 – 2.81) | 3.19, 0.21  (2.78 – 3.59) | 85.61, 13.61  (58.94 – 112.28) | 124.97, 8.10 (109.10 – 140.83) |
| Year 9 | Intervention  (n = 132) | Control  (n = 57) | Intervention  (n = 132) | Control  (n = 57) | Intervention  (n = 132) | Control  (n = 57) | Intervention  (n = 132) | Control  (n = 57) |
|  | 36.32, 0.60  (35.15 – 37.48) | 32.78, 1.59 (29.66 – 35.90) | 0.82, 0.01  (0.81 – 0.83) | 0.80, 0.00  (0.79 – 0.81) | 2.57, 0.28  (2.03 – 3.10) | 2.52, 0.79  (0.98 – 4.06) | 97.79, 15.89  (66.65 – 128.92) | 99.66, 20.19  (60.09 – 139.24) |
|  |  |  |  |  |  |  |  |  |
| Programme delivery “0” (Control – no intervention) | Intervention  (n = 0) | Control  (n = 427) | Intervention  (n = 0) | Control  (n = 427) | Intervention  (n = 0) | Control  (n = 427) | Intervention  (n = 0) | Control  (n = 427) |
|  | ---- | 41.73, 0.71 (40.33 – 43.13) | ---- | 0.84, 0.01  (0.83 – 0.85) | ---- | 2.83, 0.09  (2.66 – 3.00) | ---- | 116.59, 13.70 (89.74 – 143.45) |
| Programme delivery “1”  (Within curriculum delivery (base case)) | Intervention  (n = 227) | Control  (n = 0) | Intervention  (n = 227) | Control  (n = 0) | Intervention  (n = 227) | Control  (n = 0) | Intervention  (n = 227) | Control  (n = 0) |
|  | 45.12, 1.19  (42.78 – 47.46) | ---- | 0.84, 0.00  (0.84 – 0.84) | ---- | 2.93, 0.76  (1.45 – 4.41) | ---- | 102.08, 1.90  (98.36 – 105.81) | ---- |
| Programme delivery “2”  (Within curriculum and after-school delivery) | Intervention  (n = 207) | Control  (n = 0) | Intervention  (n = 207) | Control  (n = 0) | Intervention  (n = 207) | Control  (n = 0) | Intervention  (n = 207) | Control  (n = 0) |
|  | 40.22, 0.21  (39.81 – 40.63) | ---- | 0.81, 0.00  (0.81 – 0.81) | ---- | 2.15, 0.06  (2.03 – 2.27) | ---- | 82.99, 1.74  (79.59 – 86.40) | ---- |
| Programme delivery “3”  (Within curriculum, after-school, day trips and events delivery) | Intervention  (n = 136) | Control  (n = 0) | Intervention  (n = 136) | Control  (n = 0) | Intervention  (n = 136) | Control  (n = 0) | Intervention  (n = 136) | Control  (n = 0) |
|  | 40.73, 0.28  (40.19 – 41.27) | ---- | 0.82, 0.00  (0.82 – 0.82) | ---- | 2.72, 0.26  (2.21 – 3.23) | ---- | 107.14, 8.16  (91.14 – 123.13) | ---- |

± Marginal means, SEs and 95% Confidence Intervals all rounded to 2 decimal places.
